# The potential to expand antiretroviral therapy by improving health facility efficiency: evidence from Kenya, Uganda, and Zambia

**DOI:** 10.1186/s12916-016-0653-z

**Published:** 2016-07-20

**Authors:** Laura Di Giorgio, Mark W. Moses, Nancy Fullman, Alexandra Wollum, Ruben O. Conner, Jane Achan, Tom Achoki, Kelsey A. Bannon, Roy Burstein, Emily Dansereau, Brendan DeCenso, Kristen Delwiche, Herbert C. Duber, Emmanuela Gakidou, Anne Gasasira, Annie Haakenstad, Michael Hanlon, Gloria Ikilezi, Caroline Kisia, Aubrey J. Levine, Mashekwa Maboshe, Felix Masiye, Samuel H. Masters, Chrispin Mphuka, Pamela Njuguna, Thomas A. Odeny, Emelda A. Okiro, D. Allen Roberts, Christopher J. L. Murray, Abraham D. Flaxman

**Affiliations:** Institute for Health Metrics and Evaluation, University of Washington, 2301 5th Ave, Suite 600, Seattle, WA 98121 USA; Infectious Diseases Research Collaboration, Mulago Hospital Complex, Kampala, Uganda; African Leaders Malaria Alliance, Kampala, Uganda; Action Africa Help-International, Nairobi, Kenya; School of Humanities and Social Sciences, University of Zambia, Lusaka, Zambia; Afya Resource Associates, Nairobi, Kenya; Bill & Melinda Gates Foundation, Seattle, WA USA

**Keywords:** Antiretroviral therapy, HIV/AIDS, Efficiency, Sub-Saharan Africa

## Abstract

**Background:**

Since 2000, international funding for HIV has supported scaling up antiretroviral therapy (ART) in sub-Saharan Africa. However, such funding has stagnated for years, threatening the sustainability and reach of ART programs amid efforts to achieve universal treatment. Improving health system efficiencies, particularly at the facility level, is an increasingly critical avenue for extending limited resources for ART; nevertheless, the potential impact of increased facility efficiency on ART capacity remains largely unknown. Through the present study, we sought to quantify facility-level technical efficiency across countries, assess potential determinants of efficiency, and predict the potential for additional ART expansion.

**Methods:**

Using nationally-representative facility datasets from Kenya, Uganda and Zambia, and measures adjusting for structural quality, we estimated facility-level technical efficiency using an ensemble approach that combined restricted versions of Data Envelopment Analysis and Stochastic Distance Function. We then conducted a series of bivariate and multivariate regression analyses to evaluate possible determinants of higher or lower technical efficiency. Finally, we predicted the potential for ART expansion across efficiency improvement scenarios, estimating how many additional ART visits could be accommodated if facilities with low efficiency thresholds reached those levels of efficiency.

**Results:**

In each country, national averages of efficiency fell below 50 % and facility-level efficiency markedly varied. Among facilities providing ART, average efficiency scores spanned from 50 % (95 % uncertainty interval (UI), 48–62 %) in Uganda to 59 % (95 % UI, 53–67 %) in Zambia. Of the facility determinants analyzed, few were consistently associated with higher or lower technical efficiency scores, suggesting that other factors may be more strongly related to facility-level efficiency. Based on observed facility resources and an efficiency improvement scenario where all facilities providing ART reached 80 % efficiency, we predicted a 33 % potential increase in ART visits in Kenya, 62 % in Uganda, and 33 % in Zambia. Given observed resources in facilities offering ART, we estimated that 459,000 new ART patients could be seen if facilities in these countries reached 80 % efficiency, equating to a 40 % increase in new patients.

**Conclusions:**

Health facilities in Kenya, Uganda, and Zambia could notably expand ART services if the efficiency with which they operate increased. Improving how facility resources are used, and not simply increasing their quantity, has the potential to substantially elevate the impact of global health investments and reduce treatment gaps for people living with HIV.

**Electronic supplementary material:**

The online version of this article (doi:10.1186/s12916-016-0653-z) contains supplementary material, which is available to authorized users.

## Background

Over 29 million people were living with HIV in 2013, but due to an unprecedented global response, HIV burden has markedly declined and an estimated 19.1 million life-years have been saved by interventions such as antiretroviral therapy (ART) [1]. This success was fueled by a rapid escalation of HIV-specific development assistance for health (DAH), rising from $1.4 billion in 2000 to $10.8 billion in 2015 [2, 3]. Yet, HIV funding has plateaued since 2010 and the paradigm for ART is shifting toward long-term care, diverging from past emergency response models of care [4]. In September 2015, the World Health Organization (WHO) revised its ART guidelines [5], stipulating that everyone living with HIV should initiate ART irrespective of disease progression. This update sets universal HIV treatment as an equity-promoting goal, but also establishes nearly 21 million people eligible for ART who have yet to receive care [1, 5]. To reach these patients and sustain current ART services without a guarantee of additional funding, increasing health system efficiency has emerged as a vital pursuit for low- and middle-income countries (LMICs) [6, 7].

Scaling up health services involves a complex interplay of policy levers, as planners must balance improving access and equitable provision of care within operational and financial constraints. A focus on efficiency has become particularly attractive for expanding ART in LMICs, as increasing efficiency represents a feasible avenue for elevating service production without a proportional rise in expenditures [8, 9]. Otherwise, expanding care hinges upon more government spending, increased DAH, or heightened out-of-pocket payments for patients – and recent projections point to relatively minimal increases in government health spending by LMICs through 2040 [10]. Technical efficiency refers to the relationship between a health facility’s inputs and outputs [11], with efficient facilities defined as those using their inputs to generate the largest quantity of outputs. From an output-oriented perspective, an inefficient health facility is one where its resources are not fully maximized, leaving usable beds empty or providers seeing few patients each day. Such inefficiencies, when aggregated up through health systems, can represent millions of missed opportunities to provide care and health dollars lost. WHO estimates that up to 40 % of health spending is wasted by system inefficiencies in LMICs [12], suggesting that substantial cost-savings and service expansion could occur if improving efficiency was prioritized.

Over the last 15 years, several studies have assessed the technical efficiency of health facilities in sub-Saharan Africa [13–31], quantifying opportunities for efficiency gains across levels of care. However, past work has largely focused on one type of facility or region, which may not reflect a country’s broader health system capacity. Facilities with incomplete input and output records are often excluded, heightening the risk for biased estimates of efficiency; for instance, facilities with more complete data may benefit from higher-quality resources and facility processes [32]. To date, few studies specifically evaluate the technical efficiency of facilities that offer ART. Two recent studies measured the efficiency of facilities providing ART in Rwanda and Zambia [27, 28], but each had samples with fewer than 35 facilities. Finally, such efficiency analyses for LMICs infrequently account for the quality of outputs facilities generate [30]. This is an area of particular concern for policymakers and providers alike, as increasing healthcare production in the absence of essential medical supplies and infrastructure will neither improve patient outcomes nor overall program sustainability.

For our analysis, we used new efficiency measurement methods specifically adapted for LMICs [33] to assess the technical efficiency of health facilities in Kenya, Uganda, and Zambia. Facility data originated from the Access, Bottlenecks, Costs, and Equity (ABCE) project, a multi-country study where data were collected from stratified random samples of country facility rosters [34]. These datasets capture inputs and outputs, including ART volumes, from hundreds of facilities, and explicitly link these data to information on stocks of medical supplies and various structural characteristics. Such linkages allowed us to construct output-specific indicators of structural quality, which served as proxy for service quality [31]. Based on efficiency estimates from facilities providing ART, we predicted the potential for ART service expansion across efficiency improvement scenarios. Recognizing current evidence gaps on efficiency in LMICs, we sought to quantify the capacity for providing more ART through gains in efficiency.

## Methods

### Data

We used nationally-representative facility data from Kenya, Uganda, and Zambia [35–37]. Facility data collection occurred from September 2011 to April 2012 for Zambia, and April to November 2012 for Kenya and Uganda. Each country dataset included publicly- and privately-owned facilities across levels of care, and provided retrospective 5-year panel data for a subset of indicators (e.g., staff, patient volumes, and services provided) and cross-sectional data on facility characteristics, equipment availability, and pharmaceutical stocks. Retrospective panel data reflected fiscal years, which largely covered 2006 to 2010 for Zambia and 2007 to 2011 for Kenya and Uganda. In instances where data were incomplete for a subset of indicators, we used Amelia II software to create 50 imputed datasets. ABCE data collection and data processing are detailed elsewhere [38].

In-depth descriptions of facility sampling approaches have been previously published for Kenya, Uganda, and Zambia [39–41], and additional detail country-specific sampling strategies can be found in the Additional file 1: Appendix S1. In sum, a two-step stratified random sampling process occurred to construct nationally representative samples of health facilities for each country. The first step entailed creating a sampling frame from which subnational geographic units (districts for Uganda and Zambia, and counties for Kenya) would be drawn. For Uganda, one rural and one urban district were randomly drawn from 10 regional boundaries commonly used by household surveys in the country. For Kenya and Zambia, counties and districts were grouped by geographic performance indicators derived from previous surveys, and one subnational unit was randomly drawn from each category. The second sampling step involved sampling facilities from selected districts or counties across a range of facility types identified for each country, in accordance with their health systems. Ministry of Health facility inventories from 2011 in Kenya and Uganda, and 2010 in Zambia, served as the facility sampling frame source. For each country, this two-step sampling process resulted in 18 to 22 districts or counties selected through the district or county sampling frame, and between approximately 180 and 270 facilities selected through the facility sampling frame. For each country, a predetermined number of facilities were randomly selected from each facility type category within selected districts or counties.

The final ABCE datasets for these countries included 625 facilities and 2973 facility-years. We excluded national hospitals, specialty facilities, dental clinics, and pharmacies due to their substantive differences in services offered and production processes. To identify specialized facilities for exclusion, we used keyword searches of survey administrator comments, as well as manual examinations for facilities in the top and bottom 5 % of the efficiency spectrum. We also excluded facilities for which all 5 years of panel data were missing. For our analysis, we used a total of 395 facilities and 1900 facility-years.

We categorized facilities, inputs, and outputs into consistently-defined groups to facilitate comparisons across levels of care and countries. Facilities were grouped into categories, or platforms, based on their number of beds: 0 (no inpatient services), 1 to 15, 16 to 50, and more than 50 beds. For inputs, we used number of full-time equivalent (FTE) facility staff disaggregated by doctors, nurses, other medical personnel, and non-medical personnel as measures of labor input, and number of beds as a measure of capital. For outputs, we used the number of outpatient visits, ART visits, antenatal care visits, births, and inpatient bed-days. ART visits were comprised of the sum of pre-ART and ART visits. Due to survey differences in Zambia, we estimated ART visits using the number of ART patients seen at facilities and multiplying these values by the average number of patient visits extracted from clinical charts.

### Structural quality adjustment

To account for potential variations in service quality, we created structural quality-adjustment scores for each output. These country- and output-specific scores were calculated by determining whether each medical supply or technology was available and functional in a given facility, and then summing the total of these binary variables. The inclusion of a given output indicator was informed by clinical guidelines, physician recommendations, and whether the indicator was captured through the ABCE facility survey administered in each country. For each facility, we divided their output-specific quality sum by the highest quality value found within the country and then multiplied their annual outputs by the facility’s output-specific quality scores. Since quality scores could not be computed over time, we applied the measure retrospectively over the panel data. The facility survey used in Zambia slightly differed from the one administered in Kenya and Uganda, so the indicators included for each of Zambia’s outputs somewhat varied. Additional file 2: Appendix S2 provides the full list of indicators used for each output by country.

### Analysis

Our study was conducted in three steps, namely (1) estimating facility efficiency scores; (2) assessing facility determinants of efficiency; and (3) predicting the potential for ART expansion. We also conducted a number of sensitivity analyses, which are detailed in Additional file 3: Appendix S3.

### Estimating facility efficiency

We used a measurement approach recently designed for quantifying technical efficiency in lower-resource settings, referred to as the ensemble method (ENS). A description of the ENS model and its merits are detailed elsewhere [33], but in sum, it combines results from restricted versions of Data Envelopment Analysis (rDEA) and Stochastic Distance Function (rSDF) [11]. DEA computes the ratio of weighted outputs to weighted inputs [42], and then assigns efficiency scores to each facility relative to a frontier set by facilities with the highest ratio of outputs to inputs. The rDEA model involved placing weight restrictions for both inputs and outputs, which offered a solution to a primary drawback – arbitrary weights – of traditional DEA [43]. SDF is commonly used to estimate technical efficiency for production processes with multiple outputs (e.g., outpatients, inpatients, ART patients) [44]. rSDF, which used a Cobb-Douglas multiple-output production function, restricted variance to be greater than zero and thus allowed interpretable measurement of error. As demonstrated in an extensive simulation study [33], combining results from rDEA and rSDF (the ENS model) provided the most robust estimates of technical efficiency, particularly when the underlying production function of facilities are uncertain. Further, the ENS model helped to offset some of DEA’s largest pitfalls: its tendency to underestimate technical efficiency in contexts with a non-linear multiple-output production function and its frequent overestimation of efficiency when the distribution of efficiency is uniform. For analyzing technical efficiency of facilities in lower-resource settings, the ENS model performance was viewed as the most preferred estimation strategy [33].

For all facility-years of data, we used the ENS model to estimate efficiency scores, which were based on the median of 50 imputed datasets. In subsequent analyses, we used efficiency scores from the most recent facility-year, viewing this as a stronger measure of current capacity for service expansion than multi-year averages of efficiency.

To produce platform- and country-level averages of efficiency, we applied weights to each facility efficiency score, as derived from ABCE sampling frames.

### Assessing facility determinants of efficiency

For each country, we logit-transformed facility efficiency scores and multivariate regressions by pooling facilities across platforms and countries, and accounted for numerous facility covariates: urbanicity; ownership; electrical connectivity; hosting of administrative meetings; hosting of personnel training; the natural logarithm of reported catchment population; fraction of FTEs absent on the day of survey; and fraction of FTEs staffed by doctors, nurses, and volunteers or externally funded personnel. We also conducted bivariate regressions stratified by platform for each country.

### Predicting the potential for ART expansion

We estimated the potential for increased ART visits across efficiency improvement scenarios, such that all facilities with ART and efficiency scores below a given threshold increased their efficiency to reach that threshold. For instance, in the 50 % efficiency improvement scenario, we considered a world where facilities with ART and efficiency scores below 50 % reached 50 % efficiency. We assessed these scenarios by 10 percentage point increments, from all facilities with ART reaching at least 10 % efficiency to 100 % (all facilities are fully efficient). We then computed the potential number of additional ART visits and corresponding percentage increases in ART for each country.

For this analysis, potential for expanded ART was defined by the radial, or proportional, expansion of all facility outputs. This parameter precluded options of opening new ART clinics within facilities or output transformation (e.g., increasing ART volumes by holding inpatient services constant), both of which could additionally scale up ART services. However, these approaches require an influx or shifting of resources, and would not capture the potential for maximizing inputs at facilities that already provide ART. Instead, we sought to quantify how much ART outputs could be increased, given observed facility resources and service offerings, through gains in efficiency.

### Uncertainly analysis

To propagate uncertainty for our estimates of facility efficiency, we used bootstrap resampling, where we randomly selected one of the 50 imputed datasets, *i*, and sampled with replacement to create a bootstrap sample, *b*, with the same dimensions as *i*. Since not all facility years in *i* were represented in *b*, we ran rDEA on *i* with weight restrictions and a frontier defined by *b*. Similarly, for restricted rSDF efficiency estimation, we calculated rSDF parameters in *b* and applied them to *i* for bootstrap rSDF efficiency scores [45]. Any efficiency score estimates exceeding 100 % were assigned a value of 100 %.

For each bootstrap resampling, we calculated ENS efficiency estimates and scale-up of ART visits. In total, we used 1000 boot-strapped samples to calculate 95 % uncertainty intervals (UIs) for all estimates of technical efficiency and further expansion for ART.

All analyses were conducted in R 3.2.2 and its Benchmarking 0.26 package.

### Role of funding

This work was supported by two grants funded by the Bill & Melinda Gates Foundation: ‘Assessing the determinants of cost-effectiveness of ART and HIV prevention programs in Kenya, Uganda and select states in India’ and the ‘Disease Control Priorities Network’. The funder was not involved in data collection, analysis, or review of the final results, nor did the funder have a role in the decision to submit this manuscript for publication.

## Results

Across countries, facility inputs and outputs varied markedly (Table 1). Facility averages for staff and patient volumes generally increased alongside facility size, but within platforms, sizeable differences existed. For instance, in Zambia, the second-largest and largest facilities averaged about 7100 and 31,300 ART visits, respectively, but each platform type also featured one facility with over 80,000 ART visits.

**Table 1:** Facility descriptive statistics, by country and platform

Indicator		Kenya				Uganda				Zambia			
		0 beds	1–15 beds	16–50 beds	> 50 beds	0 beds	1–15 beds	16–50 beds	> 50 beds	0 beds	1–15 beds	16–50 beds	> 50 beds
Facility inputs	Doctors	0.1 (0–1)	0.2 (0–2)	0.9 (0–4)	20.1 (2–59)	0.4 (0–3)	0.1 (0–1)	0.6 (0–2)	13.9 (0–62)	0.2 (0–1)	0.1 (0–1)	0.4 (0–2)	5.8 (1–13)
	Nurses	2.0 (0–9)	5.2 (1–28)	9.8 (2–33)	137.9 (4–429)	4.0 (0–24)	5.5 (1–23)	11.3 (3–26)	104.6 (18–311)	5.5 (1–15)	2.5 (0–17)	6.4 (0–23)	33.7 (8–94)
	Other medical staff	1.5 (0–7)	4.2 (0–25)	7.9 (1–25)	57.4 (5–191)	2.3 (0–16)	2.1 (0–10)	6.9 (1–15)	46.3 (4–167)	7.5 (0–27)	9.0 (0–121)	14.8 (1–78)	48.5 (8–206)
	Non-medical staff	2.0 (0–9)	5.2 (0–18)	12.0 (1–31)	82.0 (21–275)	7.4 (0–48)	4.0 (0–37)	8.1 (2–29)	59.0 (6–193)	4.8 (0–15)	2.6 (0–23)	7.2 (0–23)	51.6 (15–160)
	Beds	0.0 (0–0)	7.6 (1–14)	28.1 (16–50)	279.7 (67–700)	0.0 (0–0)	7.2 (1–15)	28.3 (16–48)	227.1 (67–532)	0.0 (0–0)	6.1 (1–15)	23.2 (16–43)	153.3 (60–458)
Facility outputs	Outpatient visits	4664.0 (283–26,402)	10,735.7 (720–62,178)	11,306.9 (672–42,050)	66,452.8 (159–231,853)	12,445.9 (475–77,544)	10,513.6 (120–26,477)	25,117.0 (1635–130,604)	80,699.9 (4601–261,697)	13,654.1 (216–27,697)	10,718.8 (445–41,608)	15,402.6 (0–39,846)	22,902.6 (0–93,205)
	ANC visits	190.5 (0–2993)	961.2 (0–6821)	1196.4 (31–3821)	4,788.1 (76–12,622)	354.1 (0–10,690)	1517.9 (0–11,230)	4138.6 (128–17,658)	12,743.1 (282–86,176)	793.7 (0–3,439)	804.9 (0–6790)	1326.6 (0–5197)	1541.2 (0–4446)
	ART visits	328.1 (0–7388)	1199.3 (0–19,663)	1858.5 (0–17,245)	15,422.2 (0–94,030)	4259.0 (0–68,101)	73.9 (0–2175)	1035.5 (0–13,466)	22,491.7 (0–304,272)	5678.1 (0–79,745)	2526.4 (0–75,415)	7107.5 (0–85,960)	31,337.5 (0–88,943)
	Inpatient visits	0.0 (0–0)	174.7 (0–1323)	1,949.3 (0–7920)	49,408.7 (2954–149,208)	0.0 (0–0)	123.0 (0–2280)	2780.3 (0–15,894)	47,633.1 (176–172,859)	0.0 (0–0)	162.9 (0–1483)	963.3 (0–6310)	22,312.2 (380–63,637)
	Deliveries	7.8 (0–84)	169.6 (0–1461)	349.7 (0–1244)	2785.5 (103–9175)	0.1 (0–3)	204.2 (0–1367)	517.9 (61–2380)	3410.1 (94–8428)	2.5 (0–36)	116.8 (0–1128)	520.4 (0–3730)	1196.6 (70–3127)
Percent of facilities providing ART		14 %	31 %	46 %	65 %	13 %	82 %	32 %	83 %	29 %	15 %	36 %	89 %
Total number of facilities		37	42	28	20	40	49	28	29	17	62	25	18

Averages of facility inputs and outputs are reported by country and platform, and the range for each group is reported within parentheses. ANC, antenatal care; ART, antiretroviral therapy

**Fig. 1:**
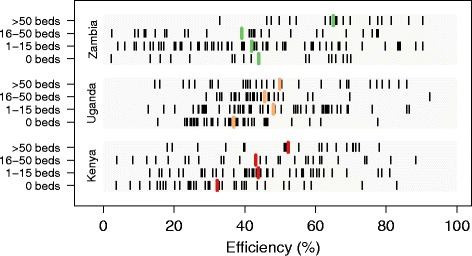
Range of facility efficiency scores, by country and platform. Note: Each black bar represents a facility’s efficiency score for the most recent year of facility data. The vertical line represents the average efficiency score across all facilities within a given platform and country

In calculating averages of technical efficiency across platforms and countries, two main findings emerged (Fig. 1): (1) average efficiency scores were relatively low and (2) each country demonstrated massive within-platform heterogeneity. Across all facilities, Uganda had the highest average efficiency score (40 %; 95 % UI, 33–47 %), followed by Zambia (39 %; 95 % UI, 37–49 %) and Kenya (34 %; 95 % UI, 30–42 %). We found that 64 % of facilities recorded efficiency scores of 50 % or lower, and 95 % of facilities fell below 80 % efficiency. Among facilities which provided ART, average efficiency scores were somewhat higher: 50 % (95 % UI, 48–62 %) in Uganda, 59 % (95 % UI, 53–67 %) in Zambia, and 51 % (95 % UI, 48–58 %) in Kenya. For facilities with ART, 45 % had efficiency scores of 50 % or lower and 94 % performed below 80 % efficiency. For each country and platform, there were a number of facilities with efficiency scores lower than 20 % and at least one facility scoring 85 % or higher. Table 2 provides detailed comparisons of efficiency by country and platform.

**Table 2:** Average efficiency scores, by country and platform

Platform	Kenya	Uganda	Zambia
	Average (95 % UI)	Average (95 % UI)	Average (95 % UI)
All facilities^a^	34 % (30–42 %)	40 % (33–47 %)	39 % (37–49 %)
0 beds	32 % (24–44 %)	37 % (25–48 %)	44 % (38–60 %)
1–15 beds	44 % (37–53 %)	48 % (34–55 %)	42 % (38–53 %)
16–50 beds	43 % (39–49 %)	46 % (41–58 %)	39 % (34–54 %)
> 50 beds	52 % (45–67 %)	50 % (47–64 %)	65 % (48–72 %)

^a^Nationally-weighted average. UI, uncertainty interval

Based on our multivariate analysis of efficiency and facility indicators (Tables 3 and 4), we found that most indicators were not significantly related to facility efficiency or did not show consistently significant associations with facility efficiency scores across countries or platforms. When stratified by platform, for instance, having a high proportion of FTEs staffed by doctors had a significant, positive association with higher levels of efficiency among facilities with zero beds, whereas the opposite was true – a significant, negative association with efficiency – among facilities with 1 to 15 beds. The log of reported catchment populations was significantly related to higher efficiency for two facility sizes (zero beds and 1 to 15 beds), while this relationship was attenuated for larger facilities. Among facilities with 1 to 15 beds and 16 to 50 beds, we found a significant, positive relationship between public ownership and higher efficiency scores. On the other hand, for facilities with 16 to 50 beds, having a high fraction of FTEs staffed by volunteers or externally funded personnel had a significant, negative relationship with facility-level efficiency. When facilities were pooled across platforms and stratified by country, a mixture of results emerged. Across countries, facilities with 1 to 15 beds had a significant, positive association with efficiency, whereas a less consistent relationship was found for facilities with 16 to 50 beds. Three facility characteristics had significant, positive associations with efficiency scores in Uganda: public ownership, log of the facility catchment population, and fraction of FTEs staffed by doctors. However, across the other countries, these indicators were neither significant nor consistently related to efficiency. In Zambia, the fraction of FTEs staffed by volunteer or externally funded personnel had a significant, negative relationship with efficiency. Regression results from our bivariate analyses were far less stable and conclusive, as detailed in Additional file 4: Appendix S4.

**Table 3:** Multivariate analyses of facility determinants of efficiency pooled by country (A) and platform (B)

Covariate		0 beds			1–15 beds			16–50 beds			> 50 beds		
		β	*P*	95 % CI	β	*P*	95 % CI	β	*P*	95 % CI	β	*P*	95 % CI
Country	Uganda												
	Kenya	0.22	0.453	(–0.40 to 0.81)	–0.05	0.794	(–0.50 to 0.36)	–0.42	0.378	(–1.40 to 0.53)	^a^	^a^	^a^
	Zambia	–0.02	0.958	(–0.80 to 0.73)	–0.28	0.287	(–0.80 to 0.24)	–1.61*	0.012	(–2.90 to –0.38)	0.53	0.692	(–2.90 to 3.98)
Facility location	Urban												
	Rural	–0.35	0.138	(–0.80 to 0.12)	–0.06	0.732	(–0.40 to 0.28)	–0.18	0.554	(–0.80 to 0.43)	0.25	0.416	(–0.50 to 1.03)
Facility ownership	Private												
	Public	0.36	0.185	(–0.20 to 0.90)	0.65*	0.004	(0.20 to 1.09)	1.98*	< 0.001	(1.00 to 2.93)	–0.79	0.092	(–1.80 to 0.20)
Facility regularly holds administrative meetings	No												
	Yes	0.11	0.689	(–0.50 to 0.68)	0.17	0.474	(–0.30 to 0.63)	0.00	0.996	(–1.60 to 1.59)	2.15*	0.013	(0.80 to 3.55)
Facility connection to functional electricity	No												
	Yes	–0.38	0.180	(–0.90 to 0.18)	–0.21	0.178	(–0.50 to 0.10)	–0.13	0.699	(–0.80 to 0.54)	0.47	0.587	(–1.70 to 2.66)
Facility holds training sessions	No												
	Yes	–0.56*	0.038	(–1.10 to –0.03)	–0.10	0.582	(–0.50 to 0.27)	–0.13	0.726	(–0.90 to 0.60)	0.24	0.545	(–0.80 to 1.27)
Log of facility catchment population		0.16*	0.015	(0.00 to 0.29)	0.25*	0.001	(0.10 to 0.39)	0.16	0.330	(–0.20 to 0.48)	–0.18	0.329	(–0.60 to 0.27)
Fraction of FTEs absent		0.75	0.140	(–0.30 to 1.74)	–0.29	0.363	(–0.90 to 0.34)	–0.92	0.149	(–2.20 to 0.34)	3.97	0.166	(–2.50 to 10.49)
Fraction of FTEs staffed by nurses		0.55	0.226	(–0.30 to 1.44)	0.10	0.802	(–0.70 to 0.87)	–0.50	0.647	(–2.70 to 1.67)	–1.91	0.279	(–6.10 to 2.32)
Fraction of FTEs staffed by doctors		3.21*	0.024	(0.40 to 5.98)	–3.85*	0.017	(–7.00 to –0.69)	–1.09	0.842	(–12.00 to 9.80)	–2.73	0.551	(–14.40 to 8.93)
Fraction of FTEs staffed by volunteer or externally funded personnel		0.03	0.912	(–0.60 to 0.62)	–0.32	0.174	(–0.80 to 0.14)	–1.11*	0.038	(–2.10 to –0.07)	–1.00	0.092	(–2.30 to 0.26)

* Statistically significant

^a^All facilities had the same value for the given covariate

CI, confidence interval; FTE, full-time equivalent

**Table 4:** Multivariate analyses of facility determinants of efficiency pooled by country (A) and platform (B)

Covariate		Kenya			Uganda			Zambia		
		β	*P*	95 % CI	β	*P*	95 % CI	β	*P*	95 % CI
Platform	0 beds									
	1–15 beds	0.51*	0.050	(0.00 to 1.01)	0.63*	< 0.001	(0.30 to 0.93)	0.63*	0.026	(0.10 to 1.19)
	16–50 beds	0.04	0.914	(–0.70 to 0.75)	0.43*	0.025	(0.10 to 0.80)	0.22	0.468	(–0.40 to 0.83)
	> 50 beds	^a^	^a^	^a^	1.50*	< 0.001	(0.70 to 2.29)	1.02*	0.048	(0.00 to 2.03)
Facility location	Urban									
	Rural	0.01	0.961	(–0.50 to 0.51)	0.03	0.864	(–0.30 to 0.34)	–0.04	0.861	(–0.60 to 0.46)
Facility ownership	Private									
	Public	0.41	0.212	(–0.20 to 1.05)	0.78*	0.002	(0.30 to 1.26)	0.57	0.056	(–0.01 to 1.16)
Facility regularly holds administrative meetings	No									
	Yes	0.17	0.570	(–0.40 to 0.77)	0.14	0.586	(–0.40 to 0.67)	0.43	0.304	(–0.40 to 1.27)
Facility connection to functional electricity	No									
	Yes	–0.17	0.640	(–0.90 to 0.56)	–0.18	0.219	(–0.50 to 0.11)	–0.33	0.138	(–0.80 to 0.11)
Facility holds training sessions	No									
	Yes	–0.46	0.172	(–1.10 to 0.20)	–0.17	0.189	(–0.40 to 0.08)	0.70	0.094	(–0.10 to 1.53)
Log of facility catchment population		0.16	0.103	(– 0.01 to 0.35)	0.22	< 0.001	(0.10 to 0.32)	0.20	0.103	(–0.02 to 0.45)
Fraction of FTEs absent		–0.94	0.105	(–2.10 to 0.20)	–0.18	0.477	(–0.70 to 0.32)	0.59	0.410	(–0.80 to 2.00)
Fraction of FTEs staffed by nurses		–0.39	0.597	(–1.80 to 1.06)	–0.03*	0.937	(–0.70 to 0.68)	0.24	0.672	(–0.90 to 1.35)
Fraction of FTEs staffed by doctors		–0.83	0.816	(–7.90 to 6.26)	3.40*	0.003	(1.20 to 5.60)	–3.92	0.068	(–8.10 to 0.29)
Fraction of FTEs staffed by volunteer or externally funded personnel		0.40	0.354	(–0.50 to 1.27)	–0.28	0.322	(–0.80 to 0.28)	–0.60*	0.047	(–1.20 to –0.01)

* Statistically significant

^a^All facilities had the same value for the given covariate

CI, confidence interval; FTE, full-time equivalent

**Fig. 2:**
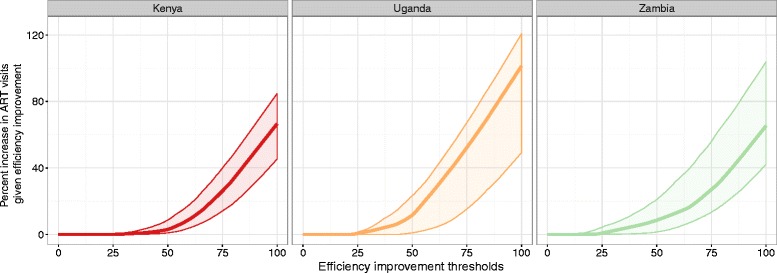
Predicted percent increases in ART visits across efficiency improvement scenarios, by country. Note: The darker line represents point estimates for predicted percent increases in ART visits, given an efficiency improvement threshold, in each country, while the shaded areas represent uncertainty intervals for point estimates. Efficiency improvement thresholds reflect the levels of technical efficiency sought by facilities with efficiency scores below the given thresholds. At the 50 % efficiency improvement threshold, all facilities with efficiency scores below 50 % would increase efficiency to 50 %; facilities with efficiency scores above 50 % would not experience increased efficiency. Percent increase in ART visits represents the predicted increase in ART visits that could be produced, given observed facility resources, if all facilities below a given efficiency improvement threshold reached that threshold. ART, antiretroviral therapy

Figure 2 illustrates the potential for ART expansion across efficiency improvement scenarios. For the scenario where all facilities with ART reached at least 50 % efficiency, we estimated a 3 % (95 % UI, 1–9 %) increase in annual ART visits in Kenya, 12 % (95 % UI, 1–23 %) in Uganda, and 9 % (95 % UI, 2–21 %) in Zambia. If all facilities with ART and efficiency scores less than 80 % reached 80 % efficiency, we estimated a 33 % (95 % UI, 19–48 %) rise in Kenya, 62 % (95 % UI, 21–78 %) in Uganda, and 33 % (95 % UI, 18–65 %) in Zambia. The latter scenario, all facilities with ART reaching 80 % efficiency, would equate to an additional 1.56 million ART visits (95 % UI, 0.871–2.25 million) in Kenya, 1.28 million (95 % UI, 0.444–1.63 million) in Uganda, and 1.77 million (95 % UI, 0.884–4.43 million) in Zambia. Table 5 details efficiency improvement scenarios and corresponding estimates of ART expansion by country. If all facilities providing ART reached at least 80 % efficiency for these countries, we estimated 459,000 new ART patients could be seen, in accordance with national recommendations on ART visit frequency during the first year of treatment [44–46].

**Table 5:** Efficiency improvement scenarios and potential increase in ART visits in Kenya, Uganda, and Zambia

Efficiency score improvement threshold	Kenya		Uganda		Zambia	
	Potential percent increase in ART visits (95 % UI)	Potential additional ART visits (95 % UI)	Potential percent increase in ART visits (95 % UI)	Potential additional ART visits (95 % UI)	Potential percent increase in ART visits (95 % UI)	Potential additional ART visits (95 % UI)
10 %	0 (0–0.005)	0 (0–243)	0 (0–0)	0 (0–0)	0 (0–0.07)	0 (0–3730)
20 %	0.02 (0.005–0.1)	1080 (222–4620)	0.03 (0–0.06)	664 (0–1270)	0.2 (0.01–2)	11,100 (710–106,000)
30 %	0.3 (0.04–1)	1270 (1830–44,100)	2 (0–4)	38,800 (0–78,300)	2 (0.2–7)	117,000 (7130–333,000)
40 %	1 (0.2–4)	50,800 (8490–168,000)	5 (0.01–12)	104,000 (253–245,000)	5 (0.5–13)	267,000 (22,400–633,000)
50 %	3 (1–9)	142,000 (45,400–405,000)	12 (1–23)	241,000 (20,500–489,000)	9 (2–21)	456,000 (76,500–1,070,000)
60 %	9 (4–18)	432,000 (186,000–843,000)	27 (4–39)	557,000 (85,000–815,000)	14 (5–33)	718,000 (234,000–1,690,000)
70 %	20 (10–32)	924,000 (463,000–1,490,000)	43 (10–57)	903,000 (218,000–1,220,000)	21 (10–48)	1,140,000 (503,000–2,470,000)
80 %	33 (19–48)	1,560,000 (871,000–2,250,000)	62 (21–78)	1,280,000 (444,000–1,630,000)	33 (18–65)	1,770,000 (884,000–3,440,000)
90 %	50 (31–66)	2,330,000 (1,460,000–3,110,000)	81 (34–99)	1,690,000 (720,000–2,090,000)	49 (29–84)	2,590,000 (1,420,000–4,430,000)
100 %	67 (45–85)	3,120,000 (2,120,000–3,970,000)	101 (49–120)	2,110,000 (1,030,000–2,550,000)	65 (42–103)	3,460,000 (2,100,000–5,440,000)

Each efficiency improvement scenario reflects the potential percent increase in ART visits and additional ART visits that health facilities could produce, given observed resources, if all health facilities with efficiency scores below a given efficiency threshold improved their efficiency score to that threshold. ART, antiretroviral therapy. UI, uncertainty interval

## Discussion

Our study showed that health facilities in Kenya, Uganda, and Zambia had relatively low technical efficiency, with each country averaging efficiency scores below 50 %. Further examination revealed massive heterogeneity, with facilities registering efficiency scores ranging from near 0 % to 95 % across levels of care. Most facility-based indicators were not significantly or consistently correlated with higher efficiency, suggesting that other characteristics, such as management practices, may be more closely linked to increased efficiency. In considering facility potential for HIV service expansion, we assessed the potential impact of efficiency gains, given observed resources at facilities providing ART, across improvement scenarios. For instance, if all facilities with ART and efficiency scores below 80 % reached 80 % efficiency, we predicted that ART visits could increase by 33 % in Kenya, 62 % in Uganda, and 33 % in Zambia. These results quantify the capacity for improved resource use and further ART scale-up in sub-Saharan Africa.

WHO recently updated its ART guidelines, recommending that everyone living with HIV initiate ART [5]. This move toward universal HIV treatment aims to significantly improve patient outcomes and curb transmission, yet, with stagnated HIV funding and millions more now eligible for ART [2, 3, 5], policymakers face tough decisions about how to pay for this influx of patients. We found that facilities in Kenya, Uganda, and Zambia could accommodate more ART initiates and continue care for established patients if their efficiency improved. In fact, if all facilities with ART reached at least 80 % efficiency in these three countries, we estimated 459,000 new ART patients could be seen at these facilities. This would represent a 40 % rise in new ART patients and progress toward reducing treatment gaps in Kenya, Uganda, and Zambia, where an estimated 2.6 million people were living with HIV and lacked ART in 2013 [1].

During the early to mid-2000s, global health initiatives sought to quickly fund and roll-out HIV services in sub-Saharan Africa [46]. These efforts, supported by a rapid escalation of DAH [2, 3], were critical to slowing the rampant spread of HIV and scaling up life-saving treatment. However, the eight-fold increase of DAH during this era has leveled off [2, 3], and HIV treatment now more closely resembles that of chronic conditions, requiring coordinated care within health systems to support both new and established ART patients. Improving how programs produce HIV services and strengthening their efficiency should now be front-and-center, particularly as the focus of both global and national policymakers shifts to maximizing HIV investments and building sustainable systems [4, 6].

Our analysis of potential determinants of efficiency could not definitively pinpoint a set of facility drivers of heightened technical efficiency across countries and platforms. Reported facility catchment population, an indicator of the population size a facility serves, was one of the only determinants that had a significant, positive relationship with higher levels of efficiency among smaller facilities. This result is not necessarily surprising, as smaller facilities (those with 15 or fewer beds) with bigger catchment populations may be located in areas where few, if no other, facilities exist to support the populations residing within them. Further, these facilities may have to accommodate more patients without experiencing a corresponding increase in medical staff, which could lead to higher efficiency scores. In many countries, including Kenya and Uganda, staffing levels are established by the Ministry of Health or other centralized health agencies [47, 48], and thus are not easily amenable to efficiency improvement, particularly at the facility level. Facility ownership, specifically public or government ownership, was another indicator for which we observed significant, positive associations with efficiency among facilities with 1 to 15 beds and 16 to 50 beds. Kenya, Uganda, and Zambia have all abolished or reduced user fees for patients seeking care at public facilities, especially for primary care and ART services [49–54]; such efforts to minimize patient medical expenses may have contributed to heightened utilization at public facilities and thus higher levels of service production. Again, however, this indicator – facility ownership – does not lend itself to facility-level initiatives to address inefficiencies.

We subsequently view more operational components of health service production as strong candidate areas for intervention at the facility level. The Institute for Healthcare Improvement emphasizes strengthening internal processes around the primary customer (patients) [55]. For instance, tailoring HIV services around ART patient stability, such as lengthening the time between ART visits for stable patients, could free facility resources for expanding HIV services or intensifying care for less stable patients [56]. Routinely collecting and reviewing data on facility operations may also help to ensure that resources optimally align with patient demand [57]. These data then can be used to more effectively deploy resources where they are needed, and identify opportunities to improve health service access [9]. A recent UNAIDS report documents the potential impact of ART efficiency gains at the facility level [6], which include using HIV “hotspot” mapping to inform facility resource allocation in Zimbabwe and integrating HIV services with other health programs in Sudan. Efforts to expand overall health system access and utilization have been a primary emphasis of past efficiency studies in sub-Saharan Africa, stressing that heightened patient demand will in turn increase facility efficiency [16, 19]. We fully agree that improving health system use has numerous benefits; nevertheless, we do not view increasing demand and facility efficiency as mutually exclusive policy decisions. Instead, our findings highlight the largely untapped potential for improving within-facility processes to extend the reach of limited resources.

Relatedly, increasing system efficiency needs to be considered alongside goals for service equity and quality [58]. Reaching maximum efficiency may not be every facility’s goal, particularly those focused on serving hard-to-reach populations or providing highly-specialized medical services. In our study, we excluded a number of these more specialized facilities, as we did not want to assess their production levels alongside less comparable facilities. Further, the view of greater technical efficiency and equity as inherently divergent health system goals is fading, particularly as plateaued HIV-specific DAH and mounting funding gaps strengthen calls for achieving ‘more with less’ against AIDS [7, 31, 59]. In fact, given current financial projections, UNAIDS stresses the need for efficiency gains to effectively reach high-risk individual and populations who currently lack ART [6]. It is also critical to ensure that facilities are not maximizing efficiency at the expense of service quality. An important avenue for improving both efficiency and quality is linking ART patient outcomes, such as viral load measures and program retention, with measures of efficiency. Future work should seek to explicitly link patient outcomes with the full range of facility inputs and outputs of interest.

### Limitations

This work should be viewed within the context of the limitations encountered, which were largely due to data issues and methodological challenges. First, facility data quality varied across countries and between facilities. We conducted extensive data cleaning procedures and used multiple imputation to limit this bias [38], but it is possible that inputs and outputs were not completely exhaustive or comparable. Second, for our analysis of efficiency determinants, we sought to include a full range of potential drivers of heightened efficiency, but some key factors, such as personnel absenteeism, may not have been adequately captured. Third, determining which specialized facilities to exclude from our analysis was largely informed by comments from survey administrators, and thus may not reflect all conceivable outliers. Fourth, our efforts to account for service quality were limited to structural facility indicators, as our datasets lacked linkages between services provided and desired patient-level outcomes such as viral load suppression. Our facility datasets also included few indicators on facility process or medical staff quality, such as competency tests, which further limited our quality adjustments primarily to structural components. In addition, this proxy quality measure was based on observations of facility supplies and equipment at the time of survey and was then applied retrospectively to our panel data. This analytic necessity may have resulted in skewed estimates of efficiency during earlier facility years, an issue that contributed to our decision to limit reported results to most recent facility-years. Fifth, we were unable to account for case mix at the facility level, an issue that may have resulted in underestimating efficiency for facilities catering to severely ill patients. We considered constructing a proxy indicator for facility case mix based on patient clinical information such as CD4 count at ART initiation, particularly since a subset of facilities in our analysis had linked, de-identified clinical chart data for ART patients [35–37]. However, due to substantive sampling and chart data limitations (e.g., in Kenya and Uganda, approximately 20 % of ART patients lacked CD4 count records at initiation in 2012 [60]), we determined that its application for the present study risked introducing notable bias. Future analyses should explore avenues for synthesizing different sources of data, including alternative facility data types or geospatial estimates, with facility assessments of efficiency.

In terms of methodological limitations, our novel approach, an ensemble model of rDEA and rSDF [33], has not been extensively tested on routine health system data. The model was developed in a simulation environment to improve on past measurement techniques and challenges posed by model choices [33]. By combining results from rDEA and rSDF, we aimed to harness each method’s strengths and offset their deficiencies [11, 33]. Second, our efficiency estimates reflected overall facility production, which may not correspond with a facility’s ART clinic efficiency levels [28]. We could not explicitly assign outputs to ART clinics within facilities, but future work should consider how efficiency may differ within facility sub-clinics. Finally, we assumed a demand for scaling up ART alongside other health services (radial expansion) and selected facility inputs, which may not necessarily reflect a country’s policy options for expanding ART or improving efficiency. For instance, program managers may target some facilities for increasing ART volumes while keeping inpatient services constant, or policymakers may introduce task-shifting initiatives to further elevate program efficiency. It is possible that these approaches, if implemented, could support greater ART expansion than what we estimated; nonetheless, our model’s parameters can be adjusted to account for production preferences.

## Conclusions

More countries have committed to achieving universal ART and global guidelines now stipulate treatment for all people living with HIV, irrespective of their disease progression. At a time when international HIV funding has stagnated and health systems are preparing to accommodate millions of newly-eligible ART patients, stretching each health dollar is vital. In applying novel methods developed to measure technical efficiency in LMICs, we found that the majority of health facilities providing ART in Kenya, Uganda, and Zambia could considerably expand ART services by increasing efficiency. Our findings emphasize the importance of how facility resources are used, rather than their sheer quantity, and how maximizing their use could notably extend the reach of life-saving treatment to all populations affected by HIV.

## Abbreviations

ABCE, Access, Bottlenecks, Costs, and Equity; ART, antiretroviral therapy; DAH, development assistance for health; ENS, ensemble method; FTE, full-time equivalent; LMICs, low- and middle-income countries; rDEA, restricted Data Envelopment Analysis; rSDF, restricted Stochastic Distance Function; UI, uncertainty interval; WHO, World Health Organization

## Additional files

Additional file 1: Appendix S1. Access, Bottlenecks, Costs, and Equity (ABCE) facility sampling strategy by country. This supplementary file details the sampling strategies adapted for Kenya (Figures A and B), Uganda (Figures C and D), and Zambia (Figures E and F) as part of the multi-country ABCE project. (DOCX 1409 kb) Additional file 2: Appendix S2. Output-specific facility indicators for quality-adjustment scores. This supplementary file provides detailed information about output-specific quality indicators used to construct structural quality-adjustment scores in Kenya, Uganda, and Zambia (Table A). (DOCX 19 kb) Additional file 3: Appendix S3. Sensitivity analyses. This supplementary file describes the sensitivity analyses used to test model performance using ART patients versus ART patient visits (Figure G and Table B), technical efficiency scores that were adjusted versus unadjusted for structural quality (Figure H and Table C), and variable approaches to estimating technical efficiency (Figure I and Table D). (DOCX 140 kb) Additional file 4: Appendix S4. Bivariate and pooled analyses of facility determinants of efficiency. Results from bivariate regressions by country (Tables E to G) and pooled across both countries and platforms (Tables H and I) are included in this supplementary file. (DOCX 40 kb)
