# Supplementary material for: The potential to expand antiretroviral therapy by improving health facility efficiency: evidence from Kenya, Uganda, and Zambia
Source: BMC Med. 2016 Jul 20;14:108. doi: 10.1186/s12916-016-0653-z (PMC4952151; doi:10.1186/s12916-016-0653-z)
Supplement: Additional file 1: Appendix S1. — Access, Bottlenecks, Costs, and Equity (ABCE) facility sampling strategy by country. This supplementary file details the sampling strategies adapted for Kenya (Figures A and B), Uganda (Figures C and D), and Zambia (Figures E and F) as part of the multi-country ABCE project. (DOCX 1409 kb) [file 12916_2016_653_MOESM1_ESM.docx]

**S1 Appendix: ABCE facility sampling strategy by country.**

**Overview**

Since its inception in 2011, the Access, Bottlenecks, Costs, and Equity (ABCE) project has sought to comprehensively identify how components of health service provision – access to services, bottlenecks in delivery, costs of care, and equity in care received – affect health system performance. Through the ABCE project, multiple sources of data, including facility surveys, are linked to together to provide a nuanced picture of how a variety of factors influence optimal health service delivery.

Existing facility data were collated and primary data collection occurred as needed in Kenya, Uganda, and Zambia. The ABCE Facility Survey was administered to nationally representative sample of health facilities in each country. To construct these nationally representative facility samples, a two-step stratified random sampling process took place for each country. In-depth descriptions of these sampling processes have been previously published [1–4]. Here we draw from these individual reports and collate these country-specific sampling procedures for Kenya, Uganda, and Zambia into one, central location.

**ABCE sample design for Kenya**

As described in the ABCE Kenya report [2], we used a two-step stratified random sampling process to create a nationally representative sample of health facilities in Kenya. Counties, from which facilities would be drawn, were grouped into 27 unique categories based on their average malnutrition rates (three categories: low [less than 20% prevalence of malnutrition], middle [20% to 30% prevalence], and high [greater than 30% prevalence of malnutrition]); health expenditures (three categories: poor [less than 20 Kenyan shillings (Kshs) per capita], middle [20 to 30 Kshs per capita], wealthy [more than 30 Kshs per capita]); and population density (three categories: rural, semi-dense, and dense). County-level estimates of malnutrition were derived from the 2005–2006 Kenya Integrated Household Budget Survey

(KIHBS). Health expenditure data were collected from the Kenya National Bureau of Statistics (KNBS) for the fiscal years of 2008/2009 and 2009/2010. Population density was determined from the 2009 census [5]. One county was randomly selected from each malnutrition-health expenditures-population category that was populated; Nairobi and Mombasa were automatically included, in addition to the randomly selected counties, due to their size and relevance to Kenya’s health service provision.

The second step, which entailed sampling facilities from each selected county, took place across the range of platforms identified in Kenya. For the ABCE project, a “platform” was defined as a channel or mechanism by which health services are delivered. Sampled health facilities included national and provincial hospitals, district and sub-district hospitals, maternity homes, health centers, dispensaries, clinics, voluntary counseling and testing (VCT) centers, and pharmacies, as well as District Health Management Teams (DHMTs). The facility sampling frame used for the ABCE project originated from the 2011 Ministry of Health (MOH) facility inventory.

A total of 18 counties were selected through the county sampling frame, and 253 facilities (excluding DHMTs) from those counties were randomly selected through the facility sampling frame:

- Up to three hospitals within the selected county, purposefully including any national hospitals.
- Up to three health centers.
- Up to three maternity homes.
- Up to two clinics, irrespective of their provision of ART.
- One clinic that offers ART.
- One dispensary, irrespective of its provision of ART.
- One dispensary that offers ART; if none existed, an additional dispensary was sampled.
- Up to two pharmacies.
- Up to one VCT center.

Within each selected county, we also included the DHMT in our sample. All national or provincial hospitals were included in the final facility sample, irrespective of their location. This means that six additional counties were included in the final ABCE sample as some national and provincial hospitals were located in a non-sampled county. However, no other facilities were selected from these non-sampled counties, as they were not drawn from the county sampling frame. Figure A displays the counties and facilities sampled for the ABCE project in Kenya, and Figure B depicts the two-step sampling process by which these counties and facilities were selected.

**Figure A. Counties and facilities sampled for the ABCE project in Kenya.**


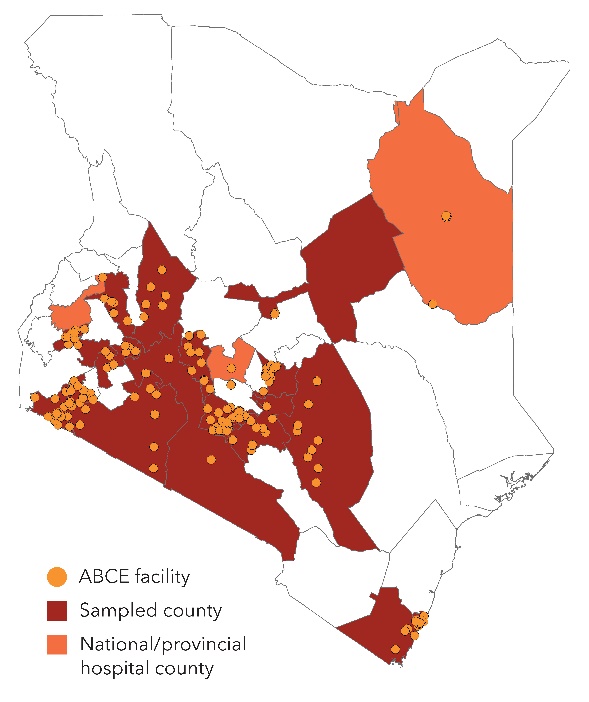


**ABCE sample design for Uganda**

As detailed in the ABCE Uganda report [3], we applied a two-step stratified random sampling process to construct a nationally representative sample of health facilities in Uganda. Districts, from which facilities would be drawn, were grouped by the ten regions designated in the 2011 Demographic and Health Survey (DHS). We randomly sampled two districts per region by urban and rural strata. Urbanicity was determined by expert input and validated by population density estimates from the 2002 Uganda Population and Housing Census [6]. The second step, which involved sampling facilities from each selected district, took place across the range of platforms identified in Uganda. Sampled health facilities included national and regional referral hospitals, district hospitals, different levels of health centers (IV,

**Figure B. Sampling strategy for the ABCE project in Kenya.**


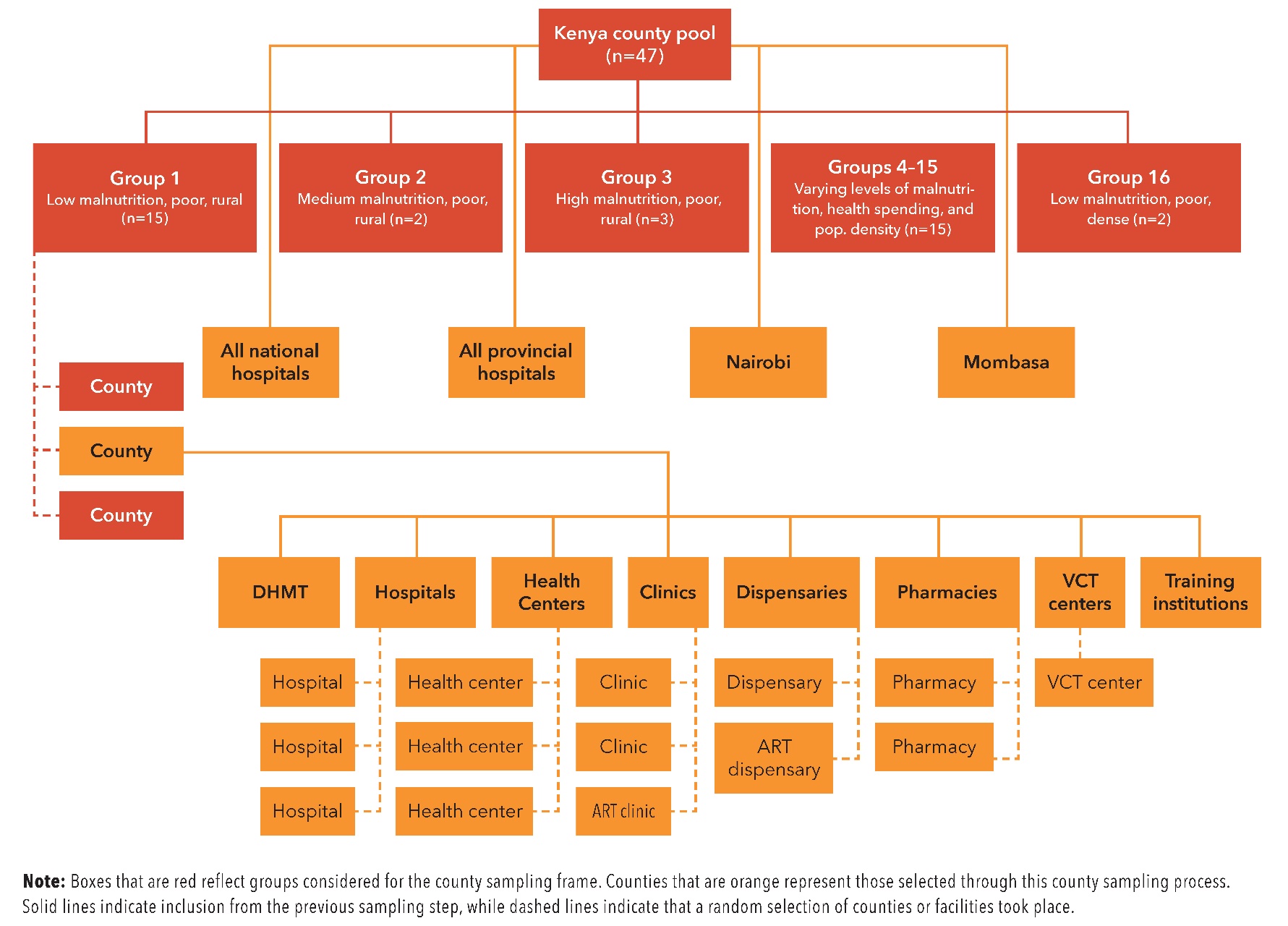


III, and II), clinics, and pharmacies or drug stores, as well as District Health Teams (DHTs). The ABCE Uganda facility sampling frame was based on the 2011 MOH facility inventory. Nineteen districts were selected through the district sampling frame (nine rural and ten urban), and 273 facilities (excluding DHTs) from those districts were selected through the facility sampling frame:

- All known hospitals within the selected district.
- All health center IVs within the selected district.
- Up to two health center IIIs that fell under the supervision of selected health center IVs.
- Up to three health center IIs that fell under the supervision of selected health center IIIs.
- Two pharmacies or drug stores.
- Up to three clinics.

Within each selected district, we also included the DHT in our sample. All national or regional referral hospitals were included in the final facility sample, irrespective of their location. However, no other facilities were selected from these non-sampled districts. Figure C shows the districts and facilities sampled in Uganda, and Figure D illustrates the two-step sampling process.

**Figure C. Districts and facilities sampled for the ABCE project in Uganda.**

**
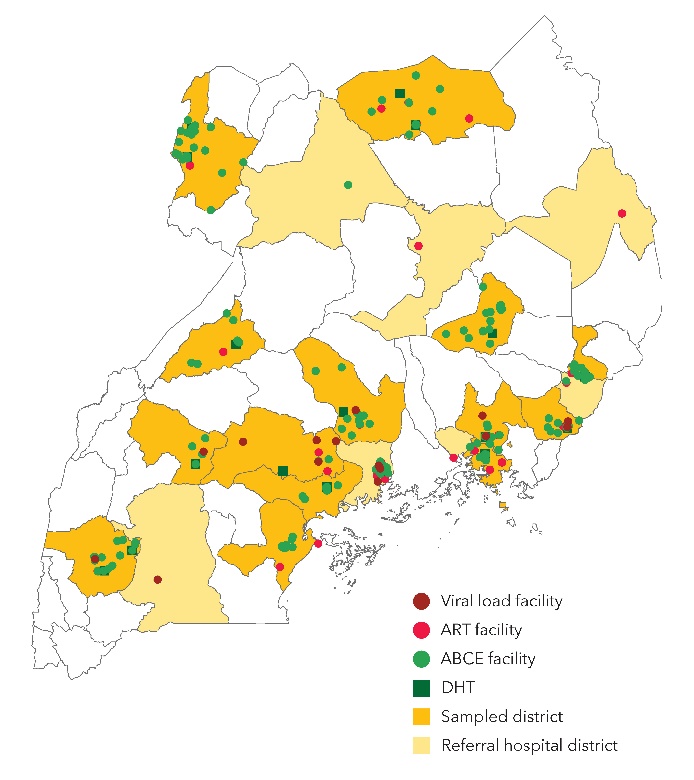
**

**ABCE sample design for Zambia**

As described in the ABCE Zambia report [4], we utilized a two-step stratified random sampling process to constructed a nationally representative sample of health facilities in Zambia. Districts, from which facilities would be drawn, were grouped into 21 unique categories based on their average levels of household wealth (poorest, middle, and wealthiest); population density (rural, semi-dense, and dense); and coverage of skilled birth attendance (SBA) (low, middle, and high). District-level estimates of household wealth and SBA were derived from the 2007 DHS. One district was randomly selected from each wealth-population-SBA category. The second step, which entailed sampling facilities from each

**Figure D. Sampling strategy for the ABCE project in Uganda.**

**
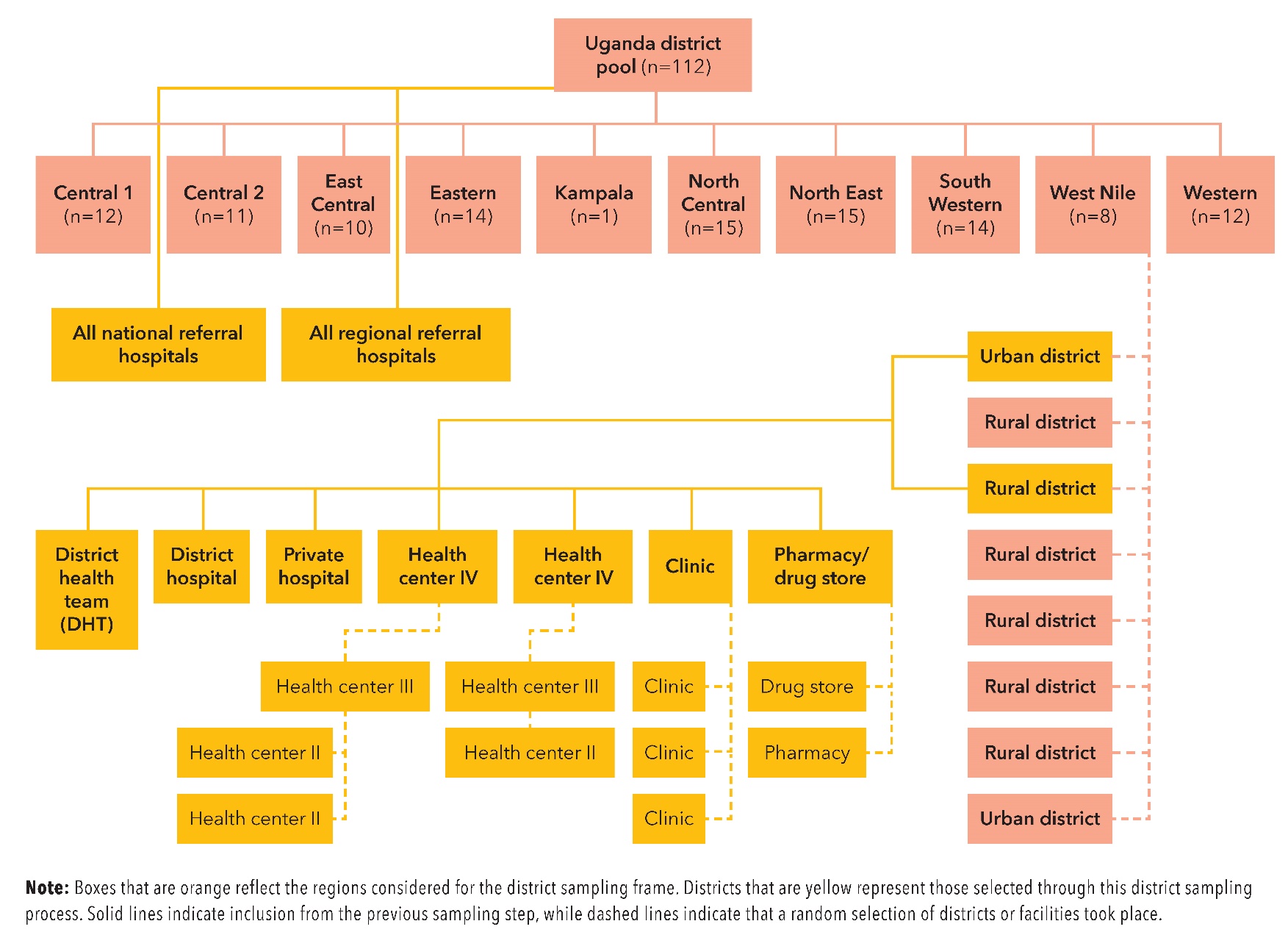
**

selected district, took place across the range of platforms in Zambia, which included level 3, level 2, and level 1 hospitals, health centers, health posts, and stores. The facility sampling frame used for the ABCE project originated from the 2010 MOH facility list. No national list of pharmacies and drug stores existed at the time of sampling, so facilities within this platform were selected using a convenience sampling approach. Twenty-two districts were selected through the district sampling frame, and 252 facilities from those districts were selected through the facility sampling frame:

- All known hospitals within the selected district.
- Two health centers within 10 kilometers of the selected district’s DHMT and three health centers located beyond 10 kilometers of the DHMT were randomly selected.
- Within a predetermined quota for districts, health posts supervised by selected rural health centers were sampled until the district quota was met.
- Two pharmacies or drug stores and one dental clinic were selected using a convenience sample.

Figure E shows the districts sampled in Zambia, and Figure F illustrates the two-step sampling process.

**Figure E. Districts sampled for the ABCE project in Zambia.**


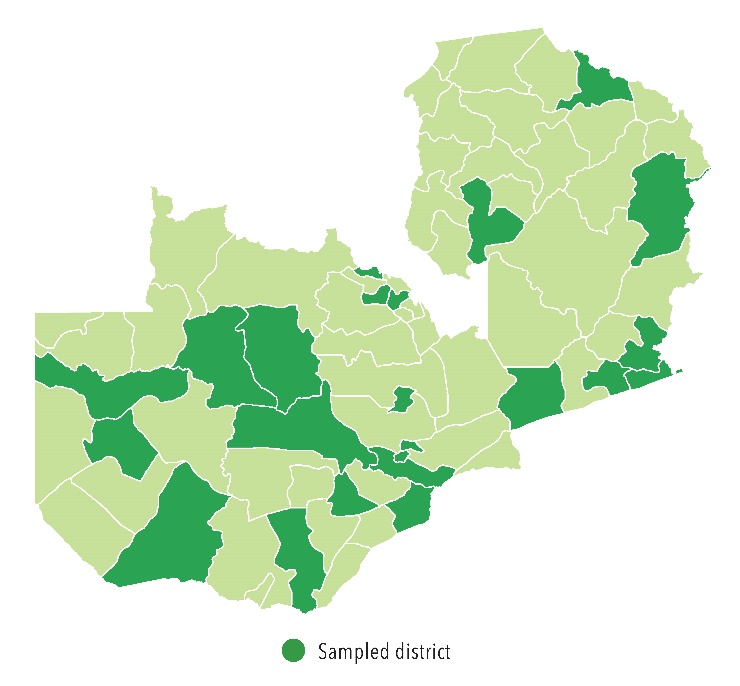


**Figure F. Sampling strategy for the ABCE project in Zambia.**


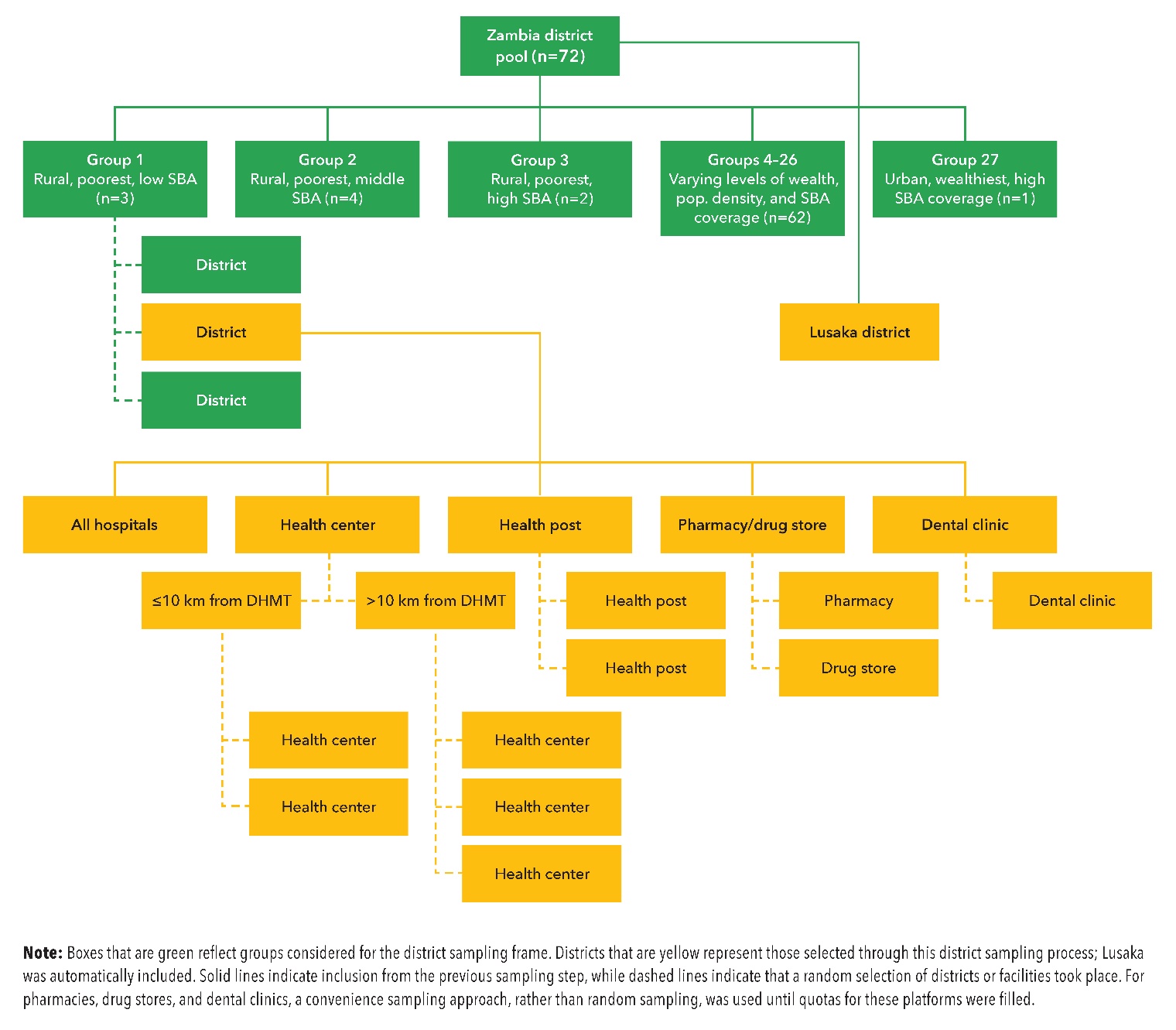


**References**

1. Institute for Health Metrics and Evaluation (IHME). Access, Bottlenecks, Costs, and Equity: ABCE Project Cross-Country Protocol. Seattle, WA: IHME; 2015.

2. Institute for Health Metrics and Evaluation (IHME). Health Service Provision in Kenya: Assessing Facility Capacity, Costs of Care, and Patient Perspectives. Seattle, WA: IHME; 2014.

3. Institute for Health Metrics and Evaluation (IHME). Health Service Provision in Uganda: Assessing Facility Capacity, Costs of Care, and Patient Perspectives. Seattle, WA: IHME; 2014.

4. Institute for Health Metrics and Evaluation (IHME). Health Service Provision in Zambia: Assessing Facility Capacity, Costs of Care, and Patient Perspectives. Seattle, WA: IHME; 2014.

5. Kenya National Bureau of Statistics (KNBS). 2009 Kenya Population Housing Census. Nairobi, Kenya: KNBS; 2010.

6. Uganda Bureau of Statistics (UBOS). 2002 Uganda Population and Housing Census: Analytical Report. Kampala, Uganda: UBOS; 2006.
