# Supplementary material for: The potential to expand antiretroviral therapy by improving health facility efficiency: evidence from Kenya, Uganda, and Zambia
Source: BMC Med. 2016 Jul 20;14:108. doi: 10.1186/s12916-016-0653-z (PMC4952151; doi:10.1186/s12916-016-0653-z)
Supplement: Additional file 2: Appendix S2. — Output-specific facility indicators for quality-adjustment scores. This supplementary file provides detailed information about output-specific quality indicators used to construct structural quality-adjustment scores in Kenya, Uganda, and Zambia (Table A). (DOCX 19 kb) [file 12916_2016_653_MOESM2_ESM.docx]

**S2 Appendix: Output-specific facility indicators for quality-adjustment scores.**

**Table A. Structural quality indicators, by output type and country.**

| **Quality indicator group** | | **Country** | |
| --- | --- | --- | --- |
|  |  | **Kenya and Uganda** | **Zambia** |
| **Outpatient visits** | Pharmaceuticals | ACTs, aspirin, albendazole, amoxicillin, atenolol, chloramphenicol, ciprofloxacin, doxycycline, erythromycin, furosemide, glibenclamide, ibuprofen, insulin, isoniazid, ketoconazole, lisinopril, lorazepam, metformin, metronidazole, paracetamol, penicillin, propranolol, quinine, salbutamol | ACTs, quinine, oral antibiotics for pneumonia, oral antibiotics for dysentery, ORS, measles vaccine, BCG vaccine, OPV, DPT vaccine, Hib vaccine, hepatitis B vaccine, pentavalent vaccine, yellow fever vaccine, tetanus vaccine, deworming, tuberculosis drug regimens (categories 1, 2, and 3), aspirin, BP-lowering medications, cholesterol lowering-medications, analgesics |
|  | Medical supplies and diagnostics | HIV test, malaria test, urinalysis, hemoglobin test, syphilis test, CBC test, liver function test, renal function test, blood glucose test strips, adult scale, child scale, BP apparatus, measuring tape, X-ray machine, ECG machine, examination table, centrifuge, stethoscope | Examination table, X-ray machine, ECG machine, hemocytometer, BP apparatus, microscope, centrifuge, refrigerator for vaccines or medication, electronic balance, stethoscope |
| **ART visits** | Pharmaceuticals | Cotrimoxazole, isoniazid, stavudine + lamivudine + nevirapine, tenofovir + emtricitabine + efavirenz, tenofovir + lamivudine + efavirenz, zidovudine + lamivudine + abacavir, zidovudine + lamivudine + nevirapine, tenofovir + lamivudine + nevirapine, zidovudine + lamivudine + efavirenz | Tenofovir + lamivudine + efavirenz, zidovudine + lamivudine + abacavir, zidovudine + lamivudine + nevirapine, tuberculosis drug regimens (categories 1, 2, and 3), septrin oral antibiotics for pneumonia, oral antibiotics for dysentery, supplementary food, deworming medications |
|  | Medical supplies and diagnostics | HIV tests, hemoglobin test, syphilis test, liver function test, renal function test, adult scale, examination table, microscope, stethoscope | Facility HIV testing availability, examination table, X-ray machine, hemocytometer, microscope, refrigerator for vaccines or medication, electronic balance, stethoscope |
|  | Other | Cotrimoxazole prophylaxis guidelines, HIV-specific treatment guidelines, isoniazid prophylaxis guidelines, nutritional guidelines | Cotrimoxazole prophylaxis guidelines, HIV-positive patient guidelines, isoniazid prophylaxis guidelines, nutritional guidelines |
| **ANC visits** | Pharmaceuticals | Insulin, metronidazole, multivitamin tablets, SP, nevirapine | Iron supplements, antenatal steroids, Fansidar/SP, tetanus vaccine |
|  | Medical supplies and diagnostics | Urinalysis, hemoglobin test, blood typing, blood glucose test strips, adult scale, BP apparatus, ultrasound | Ultrasound, BP apparatus, hemocytometer |
| **Quality indicator group** | | **Country** | |
|  |  | **Kenya and Uganda** | **Zambia** |
| **Inpatient bed-days** | Pharmaceuticals | ACTs, acetyl salicylic acid, acyclovir, albendazole, amoxicillin, atenolol, captopril, cefotaxime ceftriaxone, chloramphenicol, ciprofloxacin, cloxacillin, erythromycin, furosemide, griseofulvin, haloperidol, hydralazine, ibuprofen, insulin, isoniazid + rifampicin + pyrazinamide + ethambutol, ketoconazole, lisinopril, lorazepam, metformin, metronidazole, paracetamol, penicillin, phenobarbitone sodium, quinine, salbutamol | ACTs, quinine, gentamicin, aspirin, BP-lowering medications, cholesterol-lowering medications, analgesics, ORS, isoniazid/TB medications oral antibiotics for pneumonia, oral antibiotics for dysentery, oral antibiotics for typhoid, oral antibiotics for cholera |
|  | Medical supplies and diagnostics | HIV test (rapid and/or ELISA), malaria test (rapid and/or blood smear), urinalysis (dipstick and/or microscopy), hemoglobin test syphilis test, CBC test, liver function test, renal function test, serum electrolytes, cerebral spinal fluid test (cell count), microbiology (blood, urine and CSF cultures), child scale, BP apparatus, measuring tape, adult bag-valve-mask, X-ray machine, ECG, ultrasound, CT scan, IV catheters, eye protection-face mask | X-ray machine, vital signs monitor, ultrasound equipment, CT scan, MRI machine, ECG machine, defibrillator, kidney dialysis machine, oxygen system/cylinder, hemocytometer, wheelchair, BP apparatus, microscope, centrifuge, electronic balance, homogenizer, adult bag-valve-mask, endoscope, stethoscope, flowctyometer |
| **Births** | Pharmaceuticals | Nevirapine, hydralazine, insulin, morphine, nifedipine, paracetamol | ACTs, gentamicin, iron supplements, antenatal steroids, oxytonics (e.g., oxytocin, misprostol), analgesics, BP-lowering drugs, IUD, contraceptive injections, contraceptive implants, emergency contraceptives |
|  | Medical supplies and diagnostics | IV catheters, masks, gowns, eye protection/face mask, ultrasound, neonatal bag-valve-mask, incubator, speculum, dilation and curettage kit (or equivalent equipment), vacuum extractor, cord clamp, delivery forceps, scissors/blade to cut umbilical cord, needle holder, infant scale, child scale, BP apparatus, measuring tape, sterilization equipment (dry heat sterilizer), adult bag-valve-mask, labor table, cross-match blood test, blood glucose test strips, hemoglobin test, stethoscope, microscope, wheelchair | Incubator, vital signs monitor, ultrasound, suction machine, oxygen system/cylinder, hemocytometer, wheelchair, BP apparatus, refrigerator for vaccines or medicines, sterilization equipment (electric autoclave; dry heat sterilizer; sterilizer stove), electronic balance, adult bag-valve-mask, transport incubator, stethoscope, cauterizer, examination table (proxy for labor table) |

***Note:*** *ACTs = artemisinin-based combination therapies; BP = blood pressure;* *CBC = complete blood count; CSF = cerebrospinal fluid; CT = computerized tomography; DPT* = *diphtheria-pertussis-tetanus*; *ECG = electrocardiogram; Hib* = Haemophilus influenzae *type b*; *IUD = intrauterine device; IV = intravenous; OPV = oral polio vaccine*; *ORS = oral rehydration salts; SP = sulphadoxine/pyrimethamine*; *TB = tuberculosis.*
