# Supplementary material for: The potential to expand antiretroviral therapy by improving health facility efficiency: evidence from Kenya, Uganda, and Zambia
Source: BMC Med. 2016 Jul 20;14:108. doi: 10.1186/s12916-016-0653-z (PMC4952151; doi:10.1186/s12916-016-0653-z)
Supplement: Additional file 3: Appendix S3. — Sensitivity analyses. This supplementary file describes the sensitivity analyses used to test model performance using ART patients versus ART patient visits (Figure G and Table B), technical efficiency scores that were adjusted versus unadjusted for structural quality (Figure H and Table C), and variable approaches to estimating technical efficiency (Figure I and Table D). (DOCX 140 kb) [file 12916_2016_653_MOESM3_ESM.docx]

**S3 Appendix: Sensitivity analyses.**

**ART sensitivity analysis**

The ABCE facility survey for Zambia asked about ART patients, whereas the Kenyan and Ugandan versions of the facility surveys included questions about ART visits (both ART and pre-ART). To extrapolate ART patients to ART visits in Zambia, we used visit data extracted from patient clinical charts as a scalar to estimate the number of average ART visits per patient per year. For pre-ART patients, we used the ratio between pre-ART patients to ART patients reported by each facility.

Figure G shows our sensitivity analysis results, comparing estimates of efficiency scores when using ART patients as the facility output versus ART visits. We found that using ART visits instead of ART patients had a minimal effect on country-level efficiency estimates (Table B).

**Figure G. Comparing efficiency scores using ART visits versus ART patients.**


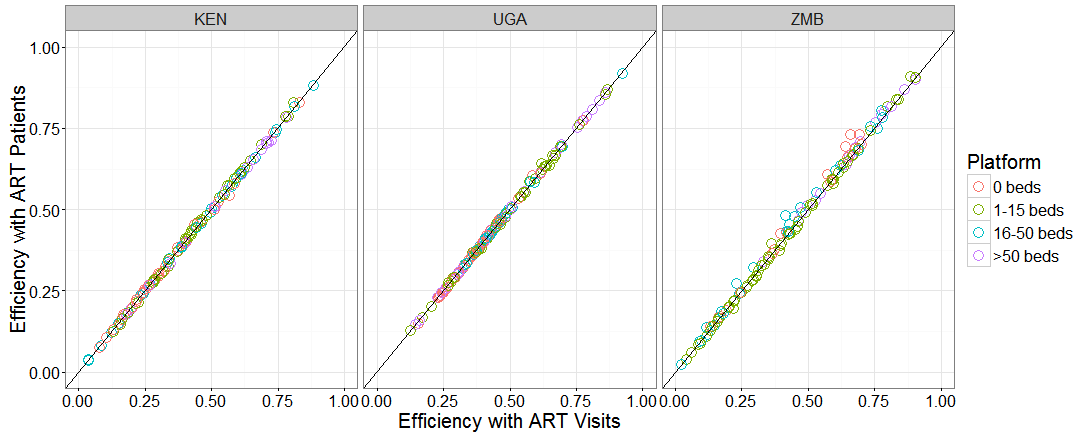


**Table B. Nationally-weighted mean efficiency scores using ART visits versus ART patients, by country.**

| **Country** | **Mean efficiency score with ART visits (95% UIs)** | **Mean efficiency score with ART patients (95% UIs)** |
| --- | --- | --- |
| Kenya | 34% (30‒42%) | 34% (30‒43%) |
| Uganda | 40% (33‒47%) | 40% (34‒47%) |
| Zambia | 39% (37‒49%) | 39% (37‒49%) |

***Note:*** *ART = antiretroviral therapy; UI = uncertainty interval.*

**Structural quality-adjustment sensitivity analysis**

In the absence of quality indicators for patient outcomes, we used output-specific structural quality-adjustment scores as a proxy. Figure H shows sensitivity analysis results comparing estimates of efficiency scores when we applied the structural quality-adjustment scores to facility outputs and those without quality adjustment. We found that the use of structural quality-adjustment indicators had a minimal effect on country-level efficiency estimates (Table C).

**Figure H. Comparing efficiency scores with and without structural quality adjustment, by country.**


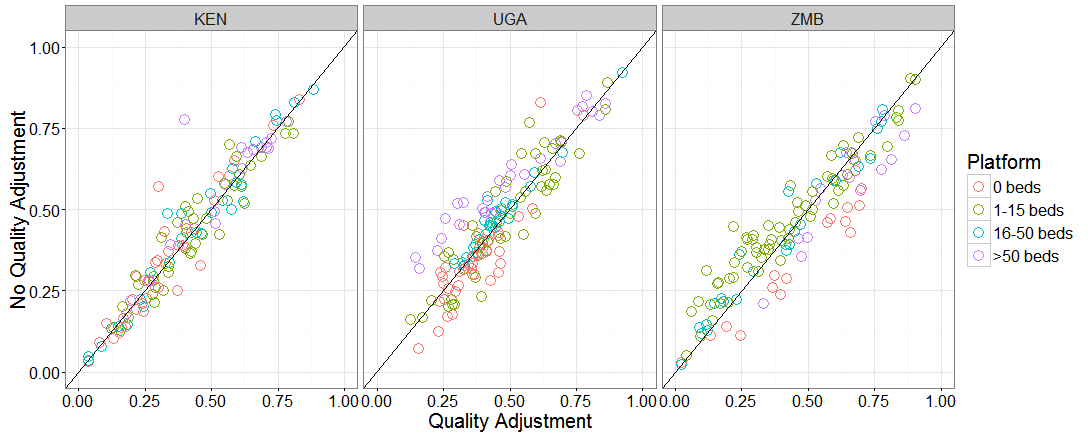


**Table C. Nationally-weighted mean efficiency scores with and without quality adjustment, by country.**

| **Country** | **Mean efficiency score with quality adjustment (95% UIs)** | **Mean efficiency score without quality adjustment (95% UIs)** |
| --- | --- | --- |
| Kenya | 34% (30‒42%) | 36% (32‒44%) |
| Uganda | 40% (33‒47%) | 40% (36‒49%) |
| Zambia | 39% (37‒49%) | 41% (39‒52%) |

***Note:*** *ART = antiretroviral therapy; UI = uncertainty interval.*

**Efficiency estimation sensitivity analysis**

We recognized the potential heterogeneity in each country’s health system structure and service production, and thus ran our efficiency estimation analyses at the country-platform level. To test the impact of this analytic approach, we also pooled all facilities across countries and ran the ensemble model on each platform.

Figure I shows our sensitivity analysis results, comparing estimates of efficiency scores when we ran the model for each country separately and when we pooled all countries together. We found that the pooled analyses led to slightly lower estimates of technical efficiency for Kenya and Uganda (Table D), whereas the nationally-weighted mean for efficiency remained the same for Zambia. From these results it is impossible to disentangle the effects of potential differences across health systems versus an increased number of facilities under comparison.

**Figure I. Comparing efficiency scores based on country-level versus pooled analyses, by country.**


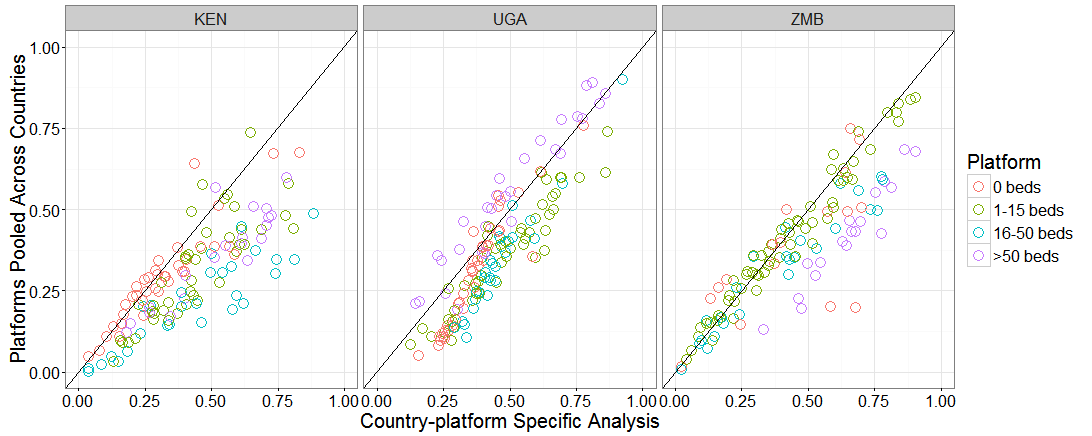


**Table D. Nationally-weighted mean efficiency scores based on country-level versus pooled analyses, by country.**

| **Country** | **Mean efficiency score with country-level analyses (95% UIs)** | **Mean efficiency score with pooled analyses (95% UIs)** |
| --- | --- | --- |
| Kenya | 34% (30‒42%) | 28% (25‒33%) |
| Uganda | 40% (33‒47%) | 31% (30‒36%) |
| Zambia | 39% (37‒49%) | 36% (34‒42%) |

***Note:*** *ART = antiretroviral therapy; UI = uncertainty interval.*
