# Supplementary material for: The potential to expand antiretroviral therapy by improving health facility efficiency: evidence from Kenya, Uganda, and Zambia
Source: BMC Med. 2016 Jul 20;14:108. doi: 10.1186/s12916-016-0653-z (PMC4952151; doi:10.1186/s12916-016-0653-z)
Supplement: Additional file 4: Appendix S4. — Bivariate and pooled analyses of facility determinants of efficiency. Results from bivariate regressions by country (Tables E to G) and pooled across both countries and platforms (Tables H and I) are included in this supplementary file. (DOCX 40 kb) [file 12916_2016_653_MOESM4_ESM.docx]

**S4 Appendix: Bivariate and pooled analyses of facility determinants of efficiency**

**Table E. Country-level bivariate analyses for Kenya**

| **Covariate** | | **Kenya** | | | | | | | | | | | |
| --- | --- | --- | --- | --- | --- | --- | --- | --- | --- | --- | --- | --- | --- |
|  |  | **0 beds** | | | **1–15 beds** | | | **16–50 beds** | | | **> 50 beds** | | |
|  |  | **β** | ***p*** | **95% CI** | **β** | ***p*** | **95% CI** | **β** | ***p*** | **95% CI** | **β** | ***p*** | **95% CI** |
| Facility location | Urban | - | - | - | - | - | - | - | - | - | - | - | - |
|  | Rural | -0.02 | 0.948 | (-0.72, 0.68) | 0.13 | 0.588 | (-0.36, 0.62) | -0.73 | 0.213 | (-1.90, 0.44) | -0.25 | 0.782 | (-2.13, 1.63) |
| Facility ownership | Private | - | - | - | - | - | - | - | - | - | - | - | - |
|  | Public | 0.62 | 0.058 | (-0.02, 1.26) | 0.21 | 0.401 | (-0.29, 0.72) | 1.62* | p < 0.001 | (0.81, 2.44) | 0.74 | 0.062 | (-0.04, 1.52) |
| Facility regularly holds administrative meetings | No | - | - | - | - | - | - | - | - | - | - | - | - |
|  | Yes | 0.23 | 0.532 | (-0.51, 0.96) | 0.29 | 0.376 | (-0.36, 0.94) | † | † | † | † | † | † |
| Facility connection to functional electricity | No | - | - | - | - | - | - | - | - | - | - | - | - |
|  | Yes | 1.15* | 0.028 | (-2.16, -0.13) | 0.15 | 0.671 | (-0.55, 0.84) | 2.71* | 0.041 | (0.12, 5.31) | † | † | † |
| Facility holds training sessions | No | - | - | - | - | - | - | - | - | - | - | - | - |
|  | Yes | 1.92* | 0.006 | (-3.26, -0.58) | 0.32 | 0.319 | (-0.32, 0.97) | -0.18 | 0.806 | (-1.67, 1.31) | -0.30 | 0.447 | (-1.13, 0.52) |
| Log of facility catchment population | | 0.10 | 0.416 | (-0.15, 0.36) | 0.08 | 0.458 | (-0.14, 0.30) | 0.30 | 0.210 | (-0.18, 0.78) | -0.01 | 0.923 | (-0.21, 0.19) |
| Fraction of FTEs absent | | -0.10 | 0.909 | (-1.91, 1.71) | -0.39 | 0.483 | (-1.49, 0.72) | -2.16 | 0.219 | (-5.82, 1.49) | ‡ | ‡ | ‡ |
| Fraction of FTEs staffed by nurses | | 0.49 | 0.537 | (-1.10, 2.07) | 0.39 | 0.609 | (-1.12, 1.89) | 1.13 | 0.588 | (-3.11, 5.36) | 1.95 | 0.158 | (-0.84, 4.74) |
| Fraction of FTEs staffed by doctors | | -0.17 | 0.961 | (-7.20, 6.87) | -4.56 | 0.302 | (-13.36, 4.25) | -5.51 | 0.500 | (-22.08, 11.06) | -3.92 | 0.587 | (-18.82, 10.97) |
| Fraction of FTEs staffed by volunteer or externally funded personnel | | 0.79 | 0.063 | (-0.05, 1.63) | 0.13 | 0.714 | (-0.59, 0.86) | 1.92* | 0.004 | (0.69, 3.16) | 0.87 | 0.071 | (-0.08, 1.82) |

***Note:*** ******* *Statistically significant. † All facilities had the same value for the given covariate. ‡ Facilities were omitted due to missingness in the fraction of FTEs absent. CI = confidence interval; FTE = full-time equivalent.*

**Table F. Country-level bivariate analyses for Uganda**

| **Covariate** | | **Uganda** | | | | | | | | | | | |
| --- | --- | --- | --- | --- | --- | --- | --- | --- | --- | --- | --- | --- | --- |
|  |  | **0 beds** | | | **1–15 beds** | | | **16–50 beds** | | | **> 50 beds** | | |
|  |  | **β** | ***p*** | **95% CI** | **β** | ***p*** | **95% CI** | **β** | ***p*** | **95% CI** | **β** | ***p*** | **95% CI** |
| Facility location | Urban | - | - | - | - | - | - | - | - | - | - | - | - |
|  | Rural | -0.04 | 0.866 | (-0.5, 0.42) | 0.25 | 0.377 | (-0.32, 0.82) | -0.37 | 0.138 | (-0.86, 0.13) | 0.26 | 0.356 | (-0.31, 0.83) |
| Facility ownership | Private | - | - | - | - | - | - | - | - | - | - | - | - |
|  | Public | 0.26 | 0.252 | (-0.19, 0.70) | 1.08* | p < 0.001 | (0.67, 1.49) | 0.66* | 0.042 | (0.03, 1.29) | 0.45 | 0.093 | (-0.08, 0.97) |
| Facility regularly holds administrative meetings | No | - | - | - | - | - | - | - | - | - | - | - | - |
|  | Yes | 0.24 | 0.646 | (-0.80, 1.27) | -0.10 | 0.840 | (-1.10, 0.90) | 0.63 | 0.314 | (-0.63, 1.90) | † | † | † |
| Facility connection to functional electricity | No | - | - | - | - | - | - | - | - | - | - | - | - |
|  | Yes | 0.18 | 0.430 | (-0.28, 0.63) | -0.43 | 0.072 | (-0.89, 0.04) | -0.35 | 0.211 | (-0.92, 0.21) | 0.13 | 0.855 | (-1.34, 1.61) |
| Facility holds training sessions | No | - | - | - | - | - | - | - | - | - | - | - | - |
|  | Yes | 0.06 | 0.780 | (-0.39, 0.52) | -0.37 | 0.122 | (-0.84, 0.10) | 0.16 | 0.556 | (-0.39, 0.70) | 1.16* | 0.019 | (0.21, 2.12) |
| Log of facility catchment population | | 0.16* | 0.001 | (0.07, 0.25) | 0.40* | p < 0.001 | (0.18, 0.61) | 0.09 | 0.457 | (-0.16, 0.35) | -0.03 | 0.493 | (-0.13, 0.07) |
| Fraction of FTEs absent | | -0.40 | 0.372 | (-1.29, 0.49) | -0.35 | 0.483 | (-1.33, 0.64) | 0.37 | 0.392 | (-0.50, 1.24) | 2.94 | 0.290 | (-5.93, 11.80) |
| Fraction of FTEs staffed by nurses | | 0.43 | 0.325 | (-0.44, 1.30) | -0.03 | 0.966 | (-1.40, 1.34) | 1.54 | 0.134 | (-0.51, 3.59) | 1.78 | 0.127 | (-0.54, 4.10) |
| Fraction of FTEs staffed by doctors | | 2.71* | 0.017 | (0.50, 4.91) | -9.56 | 0.100 | (-21.02, 1.91) | 7.97 | 0.184 | (-4.03, 19.97) | 1.03 | 0.784 | (-6.63, 8.70) |
| Fraction of FTEs staffed by volunteer or externally funded personnel | | 0.58 | 0.025 | (0.08, 1.07) | 1.07* | p < 0.001 | (0.56, 1.59) | 0.32 | 0.542 | (-0.73, 1.37) | 0.32 | 0.258 | (-0.25, 0.88) |

***Note:*** ******* *Statistically significant. † All facilities had the same value for the given covariate. ‡ Facilities were omitted due to missingness in the fraction of FTEs absent. CI = confidence interval; FTE = full-time equivalent.*

**Table G. Country-level bivariate analyses for Zambia**

| **Covariate** | | **Zambia** | | | | | | | | | | | |
| --- | --- | --- | --- | --- | --- | --- | --- | --- | --- | --- | --- | --- | --- |
|  |  | **0 beds** | | | **1–15 beds** | | | **16–50 beds** | | | **> 50 beds** | | |
|  |  | **β** | ***p*** | **95% CI** | **β** | ***p*** | **95% CI** | **β** | ***p*** | **95% CI** | **β** | ***p*** | **95% CI** |
| Facility location | Urban | - | - | - | - | - | - | - | - | - | - | - | - |
|  | Rural | -0.53 | 0.391 | (-1.82, 0.75) | -0.11 | 0.674 | (-0.65, 0.42) | 0.04 | 0.932 | (-1.03, 1.12) | 0.84* | 0.007 | (0.26, 1.43) |
| Facility ownership | Private | - | - | - | - | - | - | - | - | - | - | - | - |
|  | Public | 1.54* | 0.048 | (0.02, 3.06) | 0.81* | 0.041 | (0.04, 1.58) | 2.42* | 0.005 | (0.83, 4.01) | -0.22 | 0.526 | (-0.95, 0.50) |
| Facility regularly holds administrative meetings | No | - | - | - | - | - | - | - | - | - | - | - | - |
|  | Yes | 2.86* | 0.004 | (1.07, 4.65) | 1.07* | 0.023 | (0.15, 1.99) | 0.01 | 0.991 | (-2.62, 2.65) | 1.81* | 0.008 | (0.54, 3.08) |
| Facility connection to functional electricity | No | - | - | - | - | - | - | - | - | - | - | - | - |
|  | Yes | 0.20 | 0.807 | (-1.53, 1.94) | -0.21 | 0.370 | (-0.69, 0.26) | -0.39 | 0.485 | (-1.53, 0.75) | -0.12 | 0.876 | (-1.71, 1.47) |
| Facility holds training sessions | No | - | - | - | - | - | - | - | - | - | - | - | - |
|  | Yes | -0.07 | 0.954 | (-2.45, 2.32) | † | † | † | 0.79 | 0.391 | (-1.08, 2.66) | 0.39 | 0.266 | (-0.33, 1.11) |
| Log of facility catchment population | | 0.01 | 0.953 | (-0.36, 0.38) | 0.37* | 0.009 | (0.1, 0.65) | 0.75 | 0.125 | (-0.22, 1.72) | -0.28 | 0.221 | (-0.76, 0.20) |
| Fraction of FTEs absent | | 7.82 | 0.328 | (-8.67, 24.31) | -0.21 | 0.770 | (-1.62, 1.20) | 2.01 | 0.681 | (-7.98, 12.00) | -6.20 | 0.298 | (-18.47, 6.07) |
| Fraction of FTEs staffed by nurses | | 1.63 | 0.185 | (-0.87, 4.13) | 0.82 | 0.180 | (-0.39, 2.03) | 0.59 | 0.712 | (-2.69, 3.87) | -1.67 | 0.391 | (-5.67, 2.34) |
| Fraction of FTEs staffed by doctors | | -10.81* | 0.008 | (-18.28, -3.33) | -5.75* | 0.001 | (-9.13, -2.37) | -19.08 | 0.123 | (-43.75, 5.58) | -1.50 | 0.805 | (-14.13, 11.14) |
| Fraction of FTEs staffed by volunteer or externally funded personnel | | -0.44 | 0.584 | (-2.13, 1.24) | -0.39 | 0.252 | (-1.08, 0.29) | -0.88 | 0.212 | (-2.3, 0.54) | -0.20 | 0.822 | (-2.06, 1.66) |

***Note:*** ******* *Statistically significant. † All facilities had the same value for the given covariate. ‡ Facilities were omitted due to missingness in the fraction of FTEs absent. CI = confidence interval; FTE = full-time equivalent.*

**Table H. Multivariate analyses pooled across countries and platforms**

| **Covariate** | | **β** | ***p*** | **95% CI** |
| --- | --- | --- | --- | --- |
| Country | Uganda | - | - | - |
|  | Kenya | -0.08 | 0.631 | (-0.40, 0.23) |
|  | Zambia | -0.39* | 0.046 | (-0.80, -0.01) |
| Platform | 0 beds | - | - | - |
|  | 1‒15 beds | 0.52* | p < 0.001 | ( 0.30, 0.77) |
|  | 16‒50 beds | 0.20 | 0.194 | (-0.10, 0.50) |
|  | > 50 beds | 1.18* | p < 0.001 | ( 0.60, 1.72) |
| Facility location | Urban | - | - | - |
|  | Rural | -0.02 | 0.866 | (-0.30, 0.22) |
| Facility ownership | Private | - | - | - |
|  | Public | 0.68* | p < 0.001 | ( 0.40, 0.98) |
| Facility regularly holds administrative meetings | No | - | - | - |
|  | Yes | 0.24 | 0.180 | (-0.10, 0.59) |
| Facility connection to functional electricity | No | - | - | - |
|  | Yes | -0.17 | 0.168 | (-0.40, 0.07) |
| Facility holds training sessions | No | - | - | - |
|  | Yes | -0.10 | 0.490 | (-0.40, 0.18) |
| Log of facility catchmentpopulation | | 0.20* | p < 0.001 | ( 0.10, 0.29) |
| Fraction of FTEs absent | | -0.33 | 0.182 | (-0.80, 0.16) |
| Fraction of FTEs staffed by nurses | | 0.26 | 0.353 | (-0.30, 0.82) |
| Fraction of FTEs staffed by doctors | | 0.30 | 0.777 | (-1.80, 2.36) |
| Fraction of FTEs staffed by volunteer or externally funded personnel | | -0.28 | 0.097 | (-0.60, 0.05) |

***Note:*** ******* *Statistically significant. CI = confidence interval; FTE = full-time equivalent.*

**Table I. Bivariate analyses pooled across countries and platforms**

| **Covariate** | | **β** | ***p*** | **95% CI** |
| --- | --- | --- | --- | --- |
| Country | Uganda | - | - | - |
|  | Kenya | -0.23* | 0.050 | (-0.50, 0.00) |
|  | Zambia | -0.11 | 0.368 | (-0.30, 0.13) |
| Platform | 0 beds | - | - | - |
|  | 1‒15 beds | 0.58* | p < 0.001 | ( 0.30, 0.81) |
|  | 16‒50 beds | 0.44* | 0.001 | ( 0.20, 0.71) |
|  | > 50 beds | 1.11* | p < 0.001 | ( 0.80, 1.39) |
| Facility location | Urban | - | - | - |
|  | Rural | -0.05 | 0.605 | (-0.20, 0.14) |
| Facility ownership | Private | - | - | - |
|  | Public | 0.63* | p < 0.001 | ( 0.40, 0.82) |
| Facility regularly holds administrative meetings | No | - | - | - |
|  | Yes | 0.66* | p < 0.001 | ( 0.30, 1.01) |
| Facility connection to functional electricity | No | - | - | - |
|  | Yes | -0.08 | 0.466 | (-0.30, 0.14) |
| Facility holds training sessions | No | - | - | - |
|  | Yes | 0.26* | 0.013 | ( 0.10, 0.47) |
| Log of facility catchment population | | 0.15* | p < 0.001 | ( 0.10, 0.21) |
| Fraction of FTEs absent | | -0.07 | 0.751 | (-0.50, 0.38) |
| Fraction of FTEs staffed by nurses | | 0.54* | 0.023 | ( 0.10, 1.00) |
| Fraction of FTEs staffed by doctors | | -0.56 | 0.552 | (-2.40, 1.29) |
| Fraction of FTEs staffed by volunteer or externally funded personnel | | 0.42* | p < 0.001 | ( 0.20, 0.64) |

***Note:*** ** Statistically significant. CI = confidence interval; FTE = full-time equivalent.*
